# Treatment for Post-hemorrhagic Ventricular Dilatation: A Multiple-Treatment Meta-Analysis

**DOI:** 10.3389/fped.2020.00238

**Published:** 2020-06-23

**Authors:** Liam Mahoney, Karen Luyt, David Harding, David Odd

**Affiliations:** ^1^Neonatal Unit, North Bristol NHS Trust, Bristol, United Kingdom; ^2^Academic Department of Paediatrics, Brighton and Sussex Medical School, Brighton, United Kingdom; ^3^Bristol Medical School, University of Bristol, Bristol, United Kingdom; ^4^School of Medicine, University of Cardiff, Cardiff, United Kingdom

**Keywords:** premature birth, brain injury, preterm, intraventricular hemorrhage, post-hemorrhagic ventricular dilatation

## Abstract

**Objective:** To perform a systematic review and multiple-treatment meta-analysis for the treatment of premature infants with post-hemorrhagic ventricular dilatation (PHVD), to prevent death or long-term neuro-disability.

**Design/Method:** A systematic review was performed using PubMed, EMBASE, and the Cochrane Library. A free-word search was performed to identify likely relevant literature intervention trials of PHVD in preterm infants. Initially, network mapping was performed followed by performing a Bayesian random-effects model using the Markov chain Monte Carlo method. Areas under the cumulative ranking curve (SUCRA) were calculated as a measure of the probability that each intervention was likely to be the 1st, 2nd, 3rd, etc. best therapy. Primary outcome measure was death or moderate or severe neurodevelopmental outcome at or beyond 12 months of corrected age.

**Results:** Ten different trials were identified, enrolling 700 individuals (449 for the primary outcome). Seven intervention categories were identified, and of the 15 possible pair comparisons, 6 have been studied directly. In the multiple-treatment meta-analysis, no comparison reached conventional levels of statistical significance. Drainage Irrigation and Fibrinolytic Therapy (DRIFT) had the highest probability of being the best treatment for the primary outcome (82.1%), followed by CSF removal (10.8%), conservative management (6.7%), and then diuretic therapy (0.4%).

**Conclusions:** PHVD is a significant cause of death and disability in developed countries, yet few therapeutic options have so far been trialed. While new therapies are urgently needed for these infants, at present, NMA shows that DRIFT appears to be the most likely candidate to improve outcomes after sIVH.

## Introduction

Around 1 in 10 infants is born preterm, and survival of these infants has increased significantly over the last two decades (1, 2). However, brain injury, associated with two different patterns of damage, periventricular leukomalacia (PVL) and intraventricular hemorrhage (IVH), is common in infants born preterm, with potentially devastating personal impacts and significant population effects. While the risk of PVL in infants below 32 weeks has halved over the last two decades, the risk of IVH has stayed similar despite improvements in antenatal and neonatal care and while new interventions, such as delayed cord clamping, antenatal steroids, and intrapartum care, appear to reduce the risk of IVH, the overall risk of IVH remains high (3–6).

Consequently, it now represents the most common cause of neurological disability in the survivors (7). Those infants who develop severe intraventricular hemorrhage (sIVH) are at high risk of developing post-hemorrhagic ventricular dilatation (PHVD), a condition with high rates of long-term disability with conditions such as cerebral palsy (CP), sensory problems, and cognitive deficits (8, 9). In addition to the personal impact, the care of these premature infants who grow up with potentially disabling brain injury is expensive, with economic impacts to health care, the families involved, educational systems, and society at large (10, 11).

However, while reducing neonatal brain injury remains a research priority, no standardized treatment for these vulnerable infants appears to exist, although a number of treatments designed to prevent ongoing brain injury after sIVH and subsequent PHVD have been studied (12–26). This work aims to use Network Meta-analysis (NMA) methodology to allow the comparison of different treatment modalities for PHVD, combining the direct evidence of comparisons trailed, and deriving indirect assessments to help identify the most efficacious treatment. To our knowledge, NMA has not been applied to interventions targeted at preventing the complications and the neurodevelopmental sequelae after development of PHVD.

## Methods and Materials

### Study Selection and Data Collection

Criteria for inclusion were based on previous meta-analyses in this area and methodology was based on the Preferred Reporting Items for Systematic Reviews and Meta-Analyses guidelines (27–30). Systematic review was performed by all three authors using PubMed (1966–2018), EMBASE (1974–2018), and the Cochrane Library. A free-word search was performed (Appendix 1) to identify likely relevant literature. The search strategy was piloted to ensure that it identified studies already known by the authors to be relevant. Searches were limited to those with English language abstracts and for research in human subjects.

Three quantitative analyses were of interest: (i) death, (ii) neurodevelopmental disability, and (iii) ventriculo-peritoneal shunt surgery. The titles obtained from database searching were sifted to exclude duplicates and those clearly not relevant to the review. Abstracts of those remaining were examined and tested against the inclusion criteria.

- Randomized or quasi-randomized controlled trials for the treatment of PHVD
- At least one measure out of
  ◦ Neurodevelopmental outcomes at or beyond 12 months of age
  ◦ Mortality
  ◦ Ventriculo-peritoneal shunt surgery
- Randomized or quasi-randomized intervention trials

Full texts were obtained for those articles likely to be eligible and reviewed once more. The process was performed independently by all three authors. References in the papers were checked to identify any other possible relevant studies. Data were extracted on the characteristics of the individual studies. All three authors independently reviewed the papers identified and confirmed eligibility and the quality rating. Studies were assessed on a quality rating of adequate, unclear, or inadequate on the categories of random allocation, concealment, and blinding. Primary outcome measure was death or moderate or severe neurodevelopmental outcome at or beyond 12 months of corrected age (using any standardized measure). Where developmental outcomes were reported in a number of papers, the reported primary outcome of the study was used. An arbitrary control group was defined as treatment for PHVD only being instigated once head growth was excessive or there were clinical signs or raised intracranial pressure mandating treatment. Other interventions were grouped by consensus by two of the authors prior to analyses being performed (DO and DH). Intention-to-treat analysis was used where available. All three authors assessed the risk of bias in trials using the Cochrane risk of bias tool, with discrepancies resolved by consensus (31). Where composite outcomes of death and/or VP shunt or disability were not provided, they were calculated if possible from the presented data. Where data were unavailable, we assumed that infants who died before discharge from the neonatal unit did not receive VP shunts. Authors of included papers were not approached for missing data or where queries about a studies' methodology due to the length of time since the publications of many of the manuscripts included.

### Statistical Analysis

Network mapping was performed with the size of the nodes proportional to the sample size and the thickness of the connecting lines proportional to the number of trials. We then performed a Bayesian random-effects model using the Markov chain Monte Carlo method. The results were reported as OR with 95% confidence intervals. We used *p* < 0.05 as a conventional level of significance. The areas under the cumulative ranking curve (SUCRA) were calculated as a measure of the probability that each intervention was the best for each of the outcome measures, and for the primary outcome, we also derived the probabilities that each therapy is likely to be the 1st, 2nd, 3rd, etc. best therapy (32).

Analyses were repeated for the following outcomes:

1. Death or moderate or severe neurodevelopmental impairment at or beyond 12 months of age
2. Moderate or severe neurodevelopmental impairment at or beyond 12 months of age
3. Death in the neonatal period, or before 1 year of age
4. Requiring ventriculo-peritoneal shunt surgery in the neonatal period, or before 1 year of age
5. Requiring ventriculo-peritoneal shunt surgery or death in the neonatal period, or before 1 year of age

An assumption behind multiple-treatment meta-analysis is that direct and indirect evidence on comparisons do not disagree (coherence). To estimate this, we planned to calculate the direct and indirect OR where possible; however, due to the limited amount of studies reporting the primary outcome, we were unable to test if the direct and indirect assessments were coherent.

Finally, an *a priori* sensitivity analysis was planned excluding those studies that were considered “inadequate” on any quality measures. All analyses were performed using Stata 14. Results are presented as OR (95% confidence interval).

### Public and Patient Involvement

Patients or members of the public were not involved in the design, or conduct, reporting, or dissemination plan of this research.

## Results

### Literature Search

Databases were searched on 10/09/2018 and, after removal of duplicates, produced a list of 3445 publications. Of these, 36 abstracts were screened, and of these, a total of 11 full-text papers were reviewed, all of which fulfilled the inclusion criteria (13, 14, 16–22, 24, 25). Four systematic reviews were identified (27, 29, 33, 34) and a further three likely papers were identified from the references of the full-text papers and the existing reviews. These three additional papers were reviewed and all were included in the analysis, leaving a total of 14 papers [Appendix 2; (13–26)] from 10 different trials. The earliest publication dates were from 1980 (15), and the most recent dates were from 2019 (20). Overall, 700 individuals were randomly assigned and were included in at least one multiple-treatment meta-analysis, although only 449 infants have been enrolled in trials that have reported the primary outcome. All studies were two-armed trials. Further details are shown in Appendix 3.

Six categories of intervention were identified by the authors:

1. Control (defined as above) (13–17, 19–21, 23–26)
2. Tapping of CSF prior to symptoms or excessive head growth (16, 17)
3. Diuretic therapy (23–25)
4. Drainage Irrigation and Fibrinolytic Therapy (DRIFT) (13, 18, 19)
5. Fibrinolytic therapy (21, 22)

After discussion between authors regarding one paper randomizing infants between CSF tapping at conventional levels (2 above) and early tapping, and an additional category was added (6) (Early CSF tapping) to accommodate this intervention (20).

**Figure 1:**
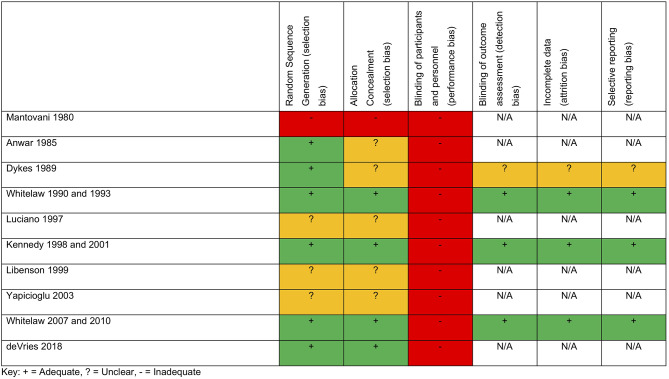
Quality measures.

A total of four studies reported an eligible neurodevelopmental measure (13, 19, 24–26), while all reported mortality or shunt usage during the neonatal period or during the first year of life. Five studies were European (13, 17, 20, 22, 24), four were based in the USA (14, 15, 23, 26), and 1 was based in Turkey (21). All studies use some form of imaging to diagnose IVH. All studies but two had a control group compatible with our pre-defined criteria; Luciano, in contrast, randomized between diuretic treatment in one arm and streptokinase in the other (22) and De Vries used two different thresholds of CSF tapping (discussed above) (20). One study (15) used an alternative number to allocate treatment arms, while a further three did not specify the randomization methods (21, 23, 35). VP shunt criteria were defined in all studies except two (15, 21). Of the four trials that reported neurodevelopmental data, three were considered adequate (13, 16, 17, 19, 24, 25) and one was unclear (26). No trial was able to blind clinicians to the intervention. A summary of quality assessments is shown in Figure 1.

### Individual Study Findings

Two studies investigated the use of furosemide and acetazolamide vs. standard treatment/LPs. Libenson (23) included a total of 16 infants who received either acetazolamide and frusemide, or daily LPs. They reported a reduction in VP shunts (10 vs. 50%) although there was crossover between treatment arms. Kennedy et al. (24, 25) also compared acetazolamide and frusemide to a control group and reported a higher rate of death and/or ventricular shunt placement in the intervention arm [RR 1.42 (1.06–1.90); *p* = 0.026] and worse developmental outcomes [e.g., RR 1.67 (1.23–2.28) for motor impairment]. Mantovani et al. (15), Dykes et al. (26), Anwar et al. (14), and the Ventriculomegaly Trial Group (16) compared frequent LP to conservative treatment, but none reported a difference in VP shunts or death between these treatment arms. De Vries et al. (20) randomized 126 infants with PHVD to intervention of repeat CSF tapping to two different thresholds with no difference in the primary outcome of VP shunt or death (*p* = 0.45). Two small studies have investigated the use of intraventricular streptokinase; Luciano et al. (22) found no difference in the rate of VP shunt between the two groups while Yapicioglu et al. (21) reported an increased need for VP shunt in the streptokinase group (83 vs. 50%). Finally, the DRIFT study included a total of 70 infants who were randomized to either intraventricular drainage, irrigation, and fibrinolytic therapy with tissue plasminogen activator (DRIFT) or to standard treatment (13). This study was closed early due a minimum chance that the short-term primary outcome would identify a difference between groups (VP shunt or death). There was no difference in the primary outcome of reducing the rates of VP shunt surgery or death but an analysis of developmental outcomes in infants at 2 years corrected age found evidence of improved neurodevelopment [adjusted OR of 0.17 (0.05–0.57)].

### Quantitative Synthesis

**Figure 2:**
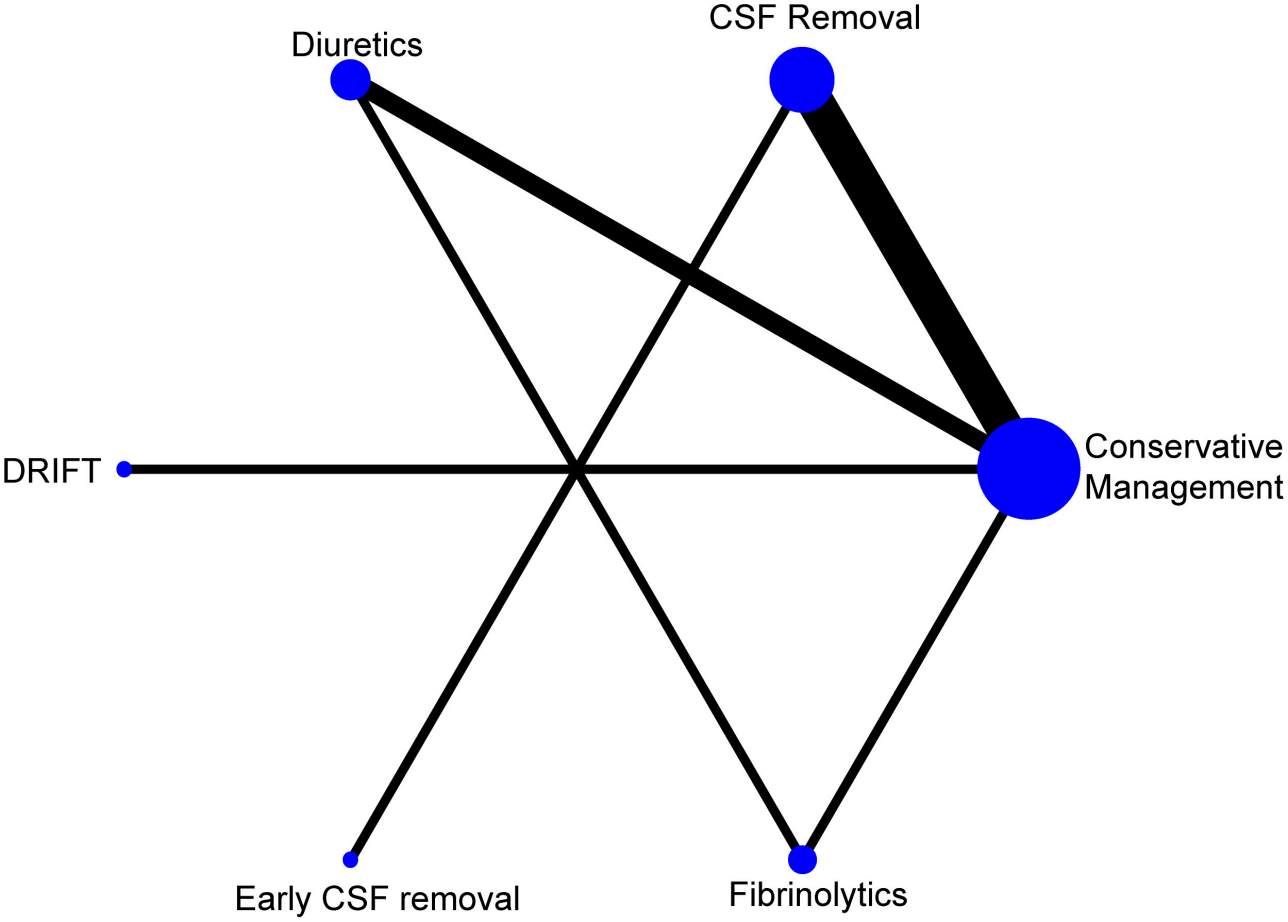
Network eligible comparisons for the multiple-treatment meta-analysis.

Figure 2 shows the resultant network of eligible comparisons from the multiple-treatment meta-analysis.

Of the 15 possible pair comparisons, 6 have been studied directly. Table 1 summarizes the results of the multiple-treatment meta-analysis for the pre-specified outcomes [e.g., compared to control diuretics, which appeared to have a higher OR of the primary outcome (OR 1.17 (0.99–1.39)], while compared to diuretics, DRIFT had a lower OR [OR 0.69 (0.44–1.07)]. Repeating the analysis for the other outcomes showed similar results. No comparison reached conventional levels of statistical significance.

**Table 1:** Comparisons between different treatment groups for direct and indirect outcome measures.

**Treatment comparison**	**Moderate or severe neuro-disability, or death**		**Moderate or severe neuro-disability**		**VP shunt or death**		**VP shunt**		**Death**	
	**OR (95% CI)**	***p***	**OR (95% CI)**	***p***	**OR (95% CI)**	***p***	**OR (95% CI)**	***p***	**OR (95% CI)**	***p***
**Compared to Control**										
CSF removal	1.01 (0.86–1.19)	0.921	0.87 (0.45–1.70)	0.689	0.96 (0.8–1.15)	0.652	0.97 (0.75–1.24)	0.791	0.91 (0.53–1.57)	0.739
Diuretics	1.17 (0.99–1.39)	0.062	1.05 (0.47–2.34)	0.905	1.20 (0.93–1.54)	0.165	1.07 (0.79–1.44)	0.676	1.58 (0.81–3.08)	0.179
DRIFT	0.80 (0.53–1.22)	0.302	0.87 (0.35–2.17)	0.759	0.97 (0.62–1.53)	0.91	1.04 (0.6–1.79)	0.89	0.58 (0.15–2.28)	0.439
Early CSF removal					0.80 (0.47–1.37)	0.421	0.80 (0.39–1.67)	0.556	0.77 (0.26–2.32)	0.645
Fibrinolytics					1.20 (0.52–2.77)	0.675	1.41 (0.70–2.84)	0.342	1.26 (0.20–7.76)	0.806
**Compared to CSF Removal**										
Diuretics	1.16 (0.92–1.47)	0.20	1.20 (0.42–3.42)	0.73	1.25 (0.91–1.70)	0.16	1.1 (0.75–1.63)	0.62	1.73 (0.73–4.10)	0.21
DRIFT	0.80 (0.51–1.24)	0.32	0.99 (0.32–3.09)	0.99	1.02 (0.62–1.65)	0.95	1.07 (0.59–1.96)	0.81	0.64 (0.15–2.78)	0.55
Early CSF removal					0.84 (0.51–1.39)	0.49	0.83 (0.42–1.65)	0.60	0.85 (0.33–2.20)	0.73
Fibrinolytics					1.25 (0.53–2.94)	0.61	1.45 (0.69–3.07)	0.33	1.38 (0.21–9.22)	0.74
**Compared to Diuretic**										
DRIFT	0.69 (0.44–1.07)	0.10	0.82 (0.24–2.79)	0.76	0.81 (0.48–1.37)	0.44	0.97 (0.52–1.82)	0.94	0.37 (0.08–1.68)	0.20
Early CSF removal					1.49 (0.37–1.21)	0.19	0.75 (0.34–1.66)	0.48	0.49 (0.14–1.77)	0.28
Fibrinolytics					1.00 (0.45–2.23)	1.00	1.32 (0.64–2.71)	0.45	0.80 (0.13–4.91)	0.81
**Compared to DRIFT**										
Early CSF removal					0.82 (0.41–1.66)	0.59	0.77 (0.31–1.92)	0.58	1.32 (0.23–7.59)	0.76
Fibrinolytics					1.23 (0.47–3.19)	0.67	1.35 (0.56–3.29)	0.51	2.15 (0.22–20.84)	0.51
**Compared to Early CSF Removal**										
Fibrinolytics					1.49 (0.55–4.04)	0.43	1.75 (0.63–4.83)	0.28	1.63 (0.19–13.62)	0.65

**Figure 3:**
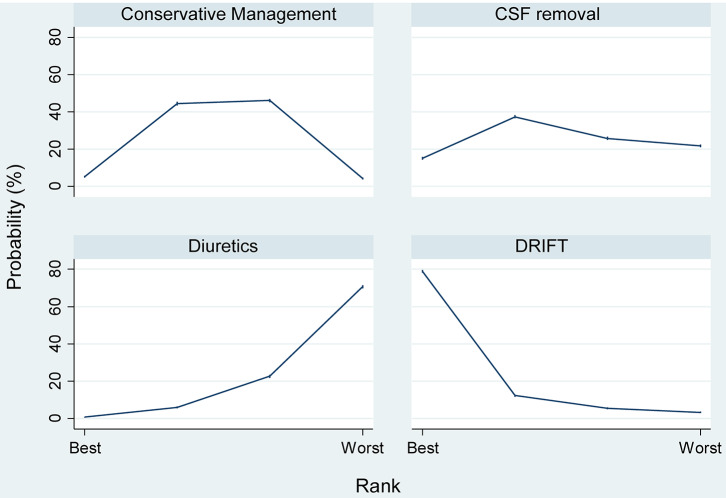
Probabilities of each therapy being the 1st, 2nd, 3rd, or 4th best treatment for the prevention of moderate/severe neuro-disability or death.

Table 2 and Figure 3 shows the SURCRA for each treatment and outcomes measured. DRIFT had the highest probability of being the best treatment for the primary outcome (82.1%), followed by CSF removal (10.8%), conservative management (6.7%), and then diuretic therapy (0.4%). No study was able to blind clinicians delivering the treatment, and so no additional analysis was performed.

**Table 2:** SUCRA for probability of most efficacious treatment for different outcomes.

**Treatment**	**Outcome**				
	**Moderate/severe neuro-disability or death**	**Moderate/severe neuro-disability**	**Death**	**VP shunt**	**VP shunt or death**
Control	0.6	0.4	0.5	0.5	0.5
CSF removal	0.5	0.6	0.6	0.6	0.6
Diuretic treatment	0.1	0.4	0.2	0.4	0.2
DRIFT	0.9	0.6	0.8	0.5	0.6
Early CSF removal	–	–	0.6	0.7	0.8
Fibrinolytics	–	–	0.4	0.2	0.3

## Discussion

We have found in this work that little evidence exists to directly differentiate any proposed or trialed therapy for PHVD. Diuretic therapy appeared to be detrimental, and ranking of therapies appeared to show that DRIFT may by the most efficacious. However, despite the significant impact of this disease, only 700 infants have ever been enrolled in an intervention trial, and further studies are desperately needed.

Limitations of this work include the heterogeneity of the infants enrolled, in part due to the wide time frame recruitment across the studies (1980–2019). Neonatal care has changed significantly during this time, with new therapies such as antenatal steroids and surfactant, and the reduction in other treatments such as postnatal steroids. Despite this, the control arm of these trials appears remarkably similar over the 40 years investigated, with VP shunt insertion after development of PHVD still the standard treatment of choice. The biggest and perhaps most influential trials have all been performed in the last 20 years and had infants of similar gestation, with similar control group interventions. We have used a random effects model to allow for increased uncertainty in this work, but the lack of precision is likely due to the small numbers included (around 700 infants involved in these randomized controlled trials) rather than heterogeneity.

The timing of intervention in each study makes the comparison of treatments in this using this methodology difficult. For example, in some of the trials, lumbar punctures were used to reduce the size of already distended ventricles compared (13) to preventing further enlargement through early intervention (23). The differences in the randomization thresholds may contribute to some of the variation seen in the outcome measured (e.g., the percentage of VP shunts required).

PHVD remains a significant problem in the developed world. There are around 8,000 preterm infants born 32 weeks gestation in England alone each year, and around 483 (6%) of these develop a sIVH (7). Overall, around 700 infants a year develop sIVH after birth, and most of these will develop some degree of motor or cognitive disability (13). In all the work reviewed here, mortality was high, and many infants who survive have complex developmental needs, a population impact likely higher than neonatal hypoxic–ischemic encephalopathy (36) or Trisomy 21 (37), with a corresponding burden to the NHS (in future healthcare needs) and society. With this in mind, we find the lack of recent trials disappointing, and this appears to be a vital area for new biomedical research.

In this work, DRIFT is likely to be the most efficacious treatment. Recent presentation of a secondary analysis of school age neurodevelopmental outcomes from this trial appears to show similar results to those used in this work on a subset of the initial trial group (38). While this recent work was a secondary analysis, the results are in parallel with those at 2 years, with children assessed at 10 years, after adjusting for gender, birthweight, and grade of IVH, with cognitive quotient being, on average, 23.47 points higher than those who received standard treatment (*p* = 0.009).

**Figure 4:**
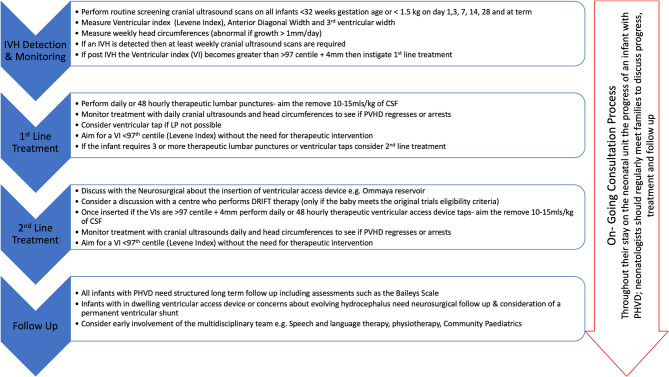
Proposed strategy for the management of PHVD.

DRIFT is however a complex intervention and involves 72 h of intensive nursing and medical care. The process of DRIFT was modified during the trial due to concerns over secondary bleeds, and the trial was stopped early due to likely futility in reaching a difference in the short-term primary outcome of shunt or death. As an alternative approach, some units have reported success with more rapid clot removal (e.g., through endoscopes) or percutaneous methods of treating intra- and extra-axial intracranial hemorrhage, although no RCT has yet been published with these novel treatments (39, 40). Equally, the ELVIS trial (20) has completed its recruitment and later neurodevelopmental outcomes are expected to be published soon. These results are eagerly awaited as a sub-study of the ELVIS trial has reported that, in the high-threshold group (LPs when the VI > p97 + 4 mm and anterior horn width >10 mm), there was more brain injury and higher ventricular volumes compared to the low-threshold groups (VI > p97 and anterior horn width >6 mm) (41). There is also interest surrounding stem cell treatment after sIVH; however, this has only been reported in phase 1 trial (42). In lieu of the results of this research, Figure 4 shows a proposed strategy for PHVD.

PHVD is a significant cause of death and disability in developed countries, yet few therapeutic options have so far been trialed. Only 700 infants have been enrolled in intervention trials, while <500 have had longer-term developmental measures reported. While new therapies are urgently needed for these infants, at present, NMA shows that DRIFT appears to be the most likely candidate to improve outcomes after sIVH, but further work is needed to implement this in routine healthcare and promising newer therapies remain untrialed.

## Data Availability Statement

The raw data supporting the conclusions of this article will be made available by the authors, without undue reservation, to any qualified researcher.

## Author Contributions

DO, DH, and LM: conception, design, collection, and assembly of data. DO, DH, LM, and KL: analysis and interpretation of the data, drafting of the article, critical revision of the article for important intellectual content and final approval.

## Conflict of Interest

The authors declare that the research was conducted in the absence of any commercial or financial relationships that could be construed as a potential conflict of interest.

## References

[1] Santhakumaran S Statnikov Y Gray D Battersby C Ashby D Modi N. Survival of very preterm infants admitted to neonatal care in England 2008-2014: time trends and regional variation. Arch Dis Child Fetal Neonatal Ed. (2018) 103:F208–15. 10.1136/archdischild-2017-312748 28883097 PMC5916099

[2] Younge N Goldstein RF Bann CM Hintz SR Patel RM Smith PB. Survival and neurodevelopmental outcomes among periviable infants. N Engl J Med. (2017) 376:617–28. 10.1056/NEJMoa1605566 28199816 PMC5456289

[3] Stoll BJ Hansen NI Bell EF Walsh MC Carlo WA Shankaran S. Trends in care practices, morbidity, and mortality of extremely preterm Neonates, 1993-2012. JAMA. (2015) 314:1039–51. 10.1001/jama.2015.10244 26348753 PMC4787615

[4] Nagano N Saito M Sugiura T Miyahara F Namba F Ota E. Benefits of umbilical cord milking versus delayed cord clamping on neonatal outcomes in preterm infants: a systematic review and meta-analysis. PLoS ONE. (2018) 13:e0201528. 10.1371/journal.pone.0201528 30161139 PMC6116944

[5] Shepherd E Salam RA Middleton P Makrides M Mcintyre S Badawi N. Antenatal and intrapartum interventions for preventing cerebral palsy: an overview of cochrane systematic reviews. Cochrane Database Syst Rev. (2017) 8:CD012077. 10.1002/14651858.CD012077.pub2 28786098 PMC6483544

[6] Gamaleldin I Harding D Siassakos D Draycott T Odd D. Significant intraventricular hemorrhage is more likely in very preterm infants born by vaginal delivery: a multi-centre retrospective cohort study. J Matern Neonatal Med. (2019) 32:477–82. 10.1080/14767058.2017.1383980 28934915

[7] Gale C Statnikov Y Jawad S Uthaya SN Modi N Modi N. Neonatal brain injuries in England: population-based incidence derived from routinely recorded clinical data held in the national neonatal research database. Arch Dis Child Fetal Neonatal Ed. (2018) 103:F301–6. 10.1136/archdischild-2017-313707 29180541 PMC6047140

[8] Radic JAE Vincer M McNeely PD. Outcomes of intraventricular hemorrhage and posthemorrhagic hydrocephalus in a population-based cohort of very preterm infants born to residents of Nova Scotia from 1993 to 2010. J Neurosurg Pediatr. (2015) 15:580–8. 10.3171/2014.11.PEDS14364 26030329

[9] Srinivasakumar P Limbrick D Munro R Mercer D Rao R Inder T. Posthemorrhagic ventricular dilatation-impact on early neurodevelopmental outcome. Am J Perinatol. (2013) 30:207–14. 10.1055/s-0032-1323581 22898993

[10] Christian EA Jin DL Attenello F Wen T Cen S Mack WJ. Trends in hospitalization of preterm infants with intraventricular hemorrhage and hydrocephalus in the United States, 2000-2010. J Neurosurg Pediatr. (2016) 17:260–9. 10.3171/2015.7.PEDS15140 26544084

[11] Kruse M Michelsen SI Flachs EM Brønnum-Hansen H Madsen M Uldall P. Lifetime costs of cerebral palsy. Dev Med Child Neurol. (2009) 51:622–8. 10.1111/j.1469-8749.2008.03190.x 19416329

[12] Department of Health and Social Care and The Rt Hon Jeremy Hunt MP New Ambition to Halve Rate of Stillbirths and Infant Deaths. London.

[13] Whitelaw A Jary S Kmita G Wroblewska J Musialik-Swietlinska E Mandera M. Randomized trial of drainage, irrigation and fibrinolytic therapy for premature infants with posthemorrhagic ventricular dilatation: developmental outcome at 2 years. Pediatrics. (2010) 125:e852–8. 10.1542/peds.2009-1960 20211949

[14] Anwar M Kadam S Hiatt IM Hegyi T. Serial lumbar punctures in prevention of post-hemorrhagic hydrocephalus in preterm infants. J Pediatr. (1985) 107:446–50. 10.1016/S0022-3476(85)80532-1 3897499

[15] Mantovani JF Pasternak JF Mathew OP Allan WC Mills MT Casper J. Failure of daily lumbar punctures to prevent the development of hydrocephalus following intraventricular hemorrhage. J Pediatr. (1980) 97:278–81. 10.1016/S0022-3476(80)80495-1 7400898

[16] Randomised trial of early tapping in neonatal posthaemorrhagic ventricular dilatation. Ventriculomegaly trial group. Arch Dis Child. (1990) 65:3–10. 10.1136/adc.65.1_spec_no.3 2407200 PMC1590164

[17] Whitelaw A Randomised trial of early tapping in neonatal posthaemorrhagic ventricular dilatation: results at 30 months. Arch Dis Child. (1994) 70:F129–36. 10.1136/fn.72.3.f211 PMC1061014 7512322

[18] Whitelaw A Pople I Cherian S Evans D Thoresen M. Phase 1 trial of prevention of hydrocephalus after intraventricular hemorrhage in newborn infants by drainage, irrigation, and fibrinolytic therapy. Pediatrics. (2003) 111(4 Pt 1):759–65. 10.1542/peds.111.4.759 12671109

[19] Whitelaw A Evans D Carter M Thoresen M Wroblewska J Mandera M. Randomized clinical trial of prevention of hydrocephalus after intraventricular hemorrhage in preterm infants: brain-washing versus tapping fluid. Pediatrics. (2007) 119:e1071–8. 10.1542/peds.2006-2841 17403819

[20] De Vries LS Groenendaal F Liem KD Heep A Brouwer AJ Van 'T Verlaat E. Treatment thresholds for intervention in posthaemorrhagic ventricular dilation: a randomised controlled trial. Arch Dis Child Fetal Neonatal Ed. (2019) 104:F70–5. 10.1136/archdischild-2017-314206 29440132

[21] Yapicioglu H Narli N Satar M Soyupak S Altunbasak S. Intraventricular streptokinase for the treatment of posthaemorrhagic hydrocephalus of preterm. J Clin Neurosci. (2003) 10:297–9. 10.1016/S0967-5868(03)00028-6 12763331

[22] Luciano R Velardi F Romagnoli C Papacci P De Stefano V Tortorolo G. Failure of fibrinolytic endoventricular treatment to prevent neonatal post haemorrhagic hydrocephalus. A case control trial. Child's Nerv Syst. (1997) 6:1. 10.1007/s003810050045 9105740

[23] Libenson MH Kaye EM Rosman NP Gilmore HE. Acetazolamide and furosemide for posthemorrhagic hydrocephalus of the newborn. Pediatr Neurol. (1999) 20:185–91. 10.1016/S0887-8994(98)00127-1 10207925

[24] Kennedy CR Ayers S Campbell MJ Elbourne D Hope P Johnson A. Randomized, controlled trial of acetazolamide and furosemide in posthemorrhagic ventricular dilation in infancy: follow-up at 1 year. Pediatrics. (2001) 108:597–607. 10.1542/peds.108.3.597 11533324

[25] Kennedy CR Campbell M Elbourne D Hope P Johnson A. International randomised controlled trial of acetazolamide and furosemide in posthaemorrhagic ventricular dilatation in infancy. Lancet. (1998) 352:433–40. 10.1016/S0140-6736(97)12390-X 9708751

[26] Dykes FD Dunbar B Lazarra A Ahmann PA. Posthemorrhagic hydrocephalus in high-risk preterm infants: natural history, management, and long-term outcome. J Pediatr. (1989) 114(4 Pt 1):611–8. 10.1016/S0022-3476(89)80707-3 2926574

[27] Whitelaw A Odd DE Intraventricular streptokinase after intraventricular hemorrhage in newborn infants. Cochrane Database Syst Rev. (2007) 17:CD000498 10.1002/14651858.CD000498.pub2 PMC7032544 17943743

[28] Whitelaw A. Repeated lumbar or ventricular punctures in newborns with intraventricular hemorrhage. Cochrane Database Syst Rev. (2017) 4:CD000216. 10.1002/14651858.cd000216 11279684

[29] Whitelaw A Brion LP Kennedy CR Odd D. Diuretic therapy for newborn infants with posthemorrhagic ventricular dilatation. Cochrane Database Syst Rev. (2001) CD002270. 10.1002/14651858.cd002270 11406041 PMC8436729

[30] Moher D Liberati A Tetzlaff J Altman DG Altman D Antes G. Preferred reporting items for systematic reviews and meta-analyses: the PRISMA statement. PLoS Med. (2009) 6:e1000097. 10.1371/journal.pmed.1000097 19621072 PMC2707599

[31] Higgins JPT Green S editors. Cochrane Handbook for Systematic Reviews of Interventions Version 5.1.0. Cochrane Collaboration (2011).

[32] Salanti G Ades AE Ioannidis JPA. Graphical methods and numerical summaries for presenting results from multiple-treatment meta-analysis: an overview and tutorial. J Clin Epidemiol. (2011) 64:163–71. 10.1016/j.jclinepi.2010.03.016 20688472

[33] Whitelaw A Lee-Kelland R. Repeated lumbar or ventricular punctures in newborns with intraventricular haemorrhage. Cochrane Database Syst Rev. (2017) 4:CD000216. 10.1002/14651858.CD000216.pub2 28384379 PMC6478098

[34] Haines SJ Lapointe M. Fibrinolytic agents in the management of posthemorrhagic hydrocephalus in preterm infants: the evidence. Child's Nerv Syst. (1999) 15:226–34. 10.1007/s003810050378 10392493

[35] Latini G Dipaola L De Felice C. First day of life reference values for pleth variability index in spontaneously breathing term newborns. Neonatology. (2012) 101:179–82. 10.1159/000331774 22024762

[36] Azzopardi D Strohm B Marlow N Brocklehurst P Deierl A Eddama O. Effects of hypothermia for perinatal asphyxia on childhood outcomes. N Engl J Med. (2014) 371:140–9. 10.1056/NEJMoa1315788 25006720

[37] Wu J Morris JK. The population prevalence of down's syndrome in England and wales in 2011. Eur J Hum Genet. (2013) 21:1016–9. 10.1038/ejhg.2012.294 23321618 PMC3746270

[38] Luyt K Jary S Lea C Young GJ Odd D Miller H. Ten-year follow-up of a randomised trial of drainage, irrigation and fibrinolytic therapy (DRIFT) in infants with post-haemorrhagic ventricular dilatation. Health Technol Assess. (2019) 23:1–116. 10.3310/hta23040 30774069 PMC6398084

[39] Etus V Kahilogullari G Karabagli H Unlu A. Early endoscopic ventricular irrigation for the treatment of neonatal posthemorrhagic hydrocephalus: a feasible treatment option or not? A multicenter study. Turk Neurosurg. (2018) 28:137–41. 10.5137/1019-5149.JTN.18677-16.0 27759873

[40] Cizmeci MN Thewissen L Zecic A Woerdeman PA Boer B Baert E. Bedside ultrasound-guided percutaneous needle aspiration of intra- and extra-axial intracranial hemorrhage in neonates. Neuropediatrics. (2018) 49:238–45. 10.1055/s-0038-1641568 29689584

[41] Cizmeci MN Khalili N Claessens NHP Groenendaal F Liem KD Heep A. Assessment of brain injury and brain volumes after posthemorrhagic ventricular dilatation: a nested substudy of the randomized controlled ELVIS trial. J Pediatr. (2019) 208:191–7.e2. 10.1016/j.jpeds.2018.12.062 30878207

[42] Ahn SY Chang YS Sung SI Park WS. Mesenchymal stem cells for severe intraventricular hemorrhage in preterm infants: phase i dose-escalation clinical trial. Stem Cells Transl Med. (2018) 7:847–56. 10.1002/sctm.17-0219 30133179 PMC6265626

