# Supplementary material for: Treatment for Post-hemorrhagic Ventricular Dilatation: A Multiple-Treatment Meta-Analysis
Source: Front Pediatr. 2020 Jun 23;8:238. doi: 10.3389/fped.2020.00238 (PMC7324764; doi:10.3389/fped.2020.00238)
Supplement: Supplementary file 1 [file Data_Sheet_1.pdf]

Appendix 1. Search strategy (Title)

1. (hydroceph* OR IVH OR intracra* OR intraventric* OR periventricul* OR ventricular* OR PHVD OR PHH OR posthaem* OR posthem* OR post-haem* OR post-hem*)
2. (newborn OR premature OR "low birth weight" OR infan* OR neonat* OR preterm)
3. random* OR trial OR placebo OR RCT
4. 1 AND 2 AND 3
5. limit 4 to (humans and english language)
