# Supplementary figures and images for: Treatment for Post-hemorrhagic Ventricular Dilatation: A Multiple-Treatment Meta-Analysis
Source: Front Pediatr. 2020 Jun 23;8:238. doi: 10.3389/fped.2020.00238 (PMC7324764; doi:10.3389/fped.2020.00238)

## Appendix 2. Results of Literature Search

### PRISMA 2009 Flow Diagram

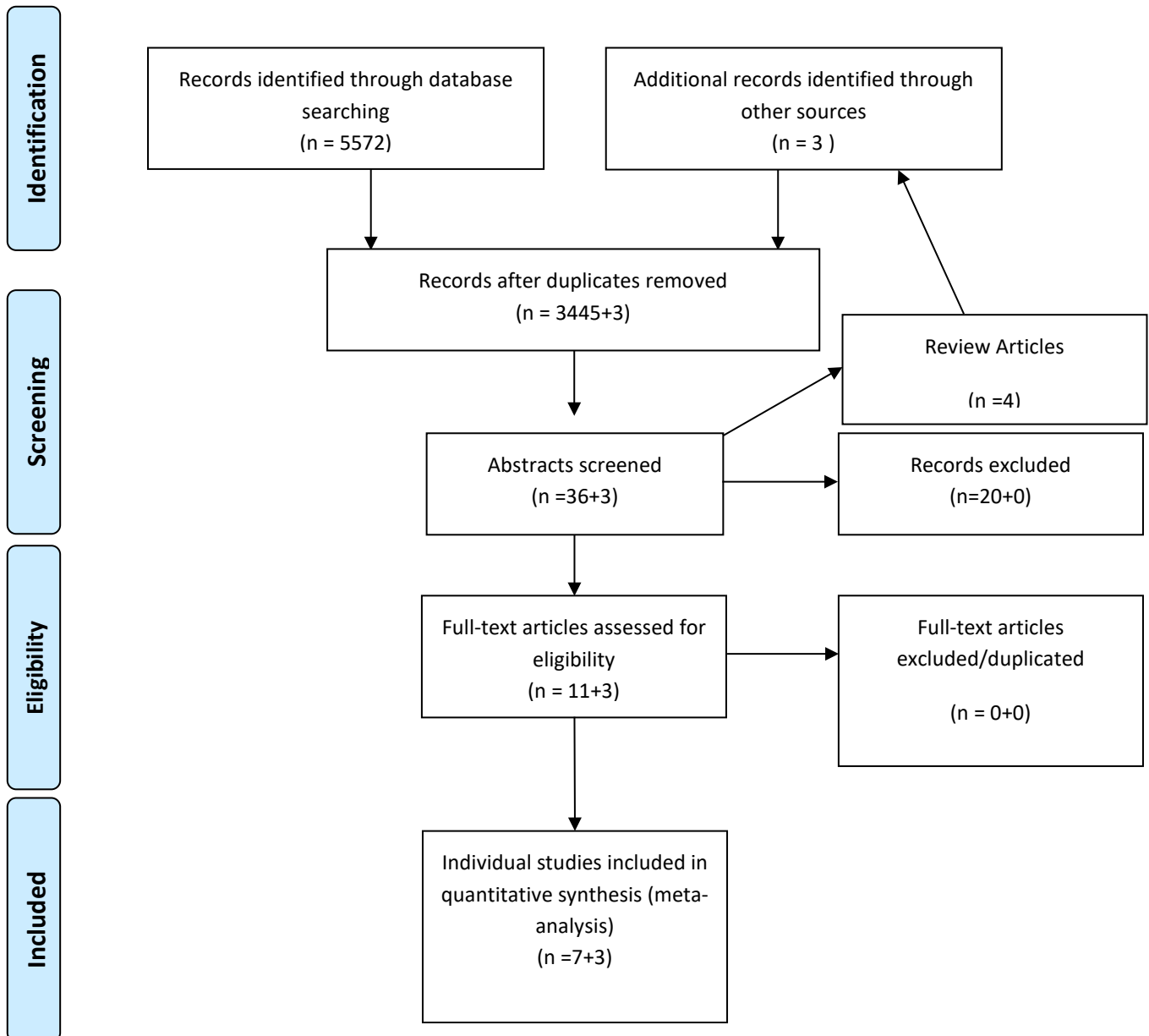

Supplement: Supplementary file 2 [file Data_Sheet_2.pdf]
