# Supplementary material for: Treatment for Post-hemorrhagic Ventricular Dilatation: A Multiple-Treatment Meta-Analysis
Source: Front Pediatr. 2020 Jun 23;8:238. doi: 10.3389/fped.2020.00238 (PMC7324764; doi:10.3389/fped.2020.00238)
Supplement: Supplementary file 3 [file Data_Sheet_3.pdf]

Appendix 3. Included Studies

Study	Years Enrolled	Country	Criteria	Randomisation	Blinding	Intervention#1	Intervention#2	Criteria for VP shunt	Neuro-disability Measure	Total Enrolled
Mantovani 1980	1978-1979	USA	Birthweight <2000g<br>IVH on CT showing haemorrhage >10% of ventricular area	Alternate placed in treatment groups	Nil	Daily lumbar punctures	Nil	No specified	None	38
Anwar 1985	1982-1984	USA	Birth weight <1500g or 1500-2500g and ventilated<br>IVH grade 3 or 4	Random numbers table	Nil	LP's to control ventricular size (up to daily)	Nil	Progressive hydrocephalus	None	47
Dykes 1989	1977-1985	USA	Gestation <35 weeks<br>No major congenital malformations,<br>Grade 3 or 4 IVH<br>Asymptomatic progressive ventricular dilation	Random number table	Not specified	Daily LPs were performed for a minimum of 1 week and a maximum of 3 weeks.	LPs only if increase in head circumference, progressive PHVD, symptomatic raised ICP or decrease in cortical mantel	No regression of PHVD and an occipital cortical mantle of ≤1 cm at 3 postnatal months.	The Bayley Scales of Infant Development<br>Stanford-Binet and Peabody Picture Vocabulary Test<br>Kaufman Assessment Battery for Children.	38
Whitelaw 1990 and 1993	1984-1987	UK	PHVD with VI >97th centile+4mm	Remote telephone randomisation<br>Balanced blocks of 6<br>Stratified by centre and grade of IVH	Neuro-developmental measurement only	LP or ventricular puncture taps to maintain ventricular size.	Nil	Failure to control head growth	Griffiths Score<br>Reynell Language Scale	157
Luciano 1997	1992-1994	Italy	PHVD with VI>97th+4mm	Randomisation mechanism not specified	Not specified	Endo-ventricular streptokinase infusion +intermittent drainage CSF to maintain normal ICP	Diuretics (furosemide 2 mg/kg/day)	Hypertensive hydrocephalus not controlled by intermittent drainage	None	12
Kennedy 1998 and 2001	1992-1996	UK	VI>97th+4mm	Computerized minimization to balance referral center and parenchymal lesions	Neuro-developmental measurement only	Acetazolamide (up to 100mg/kg/d) and Furosemide (1mg/kg/d)	LP or ventricular puncture taps only if raised ICP or excessive head growth	Two of;<br>Head size at least 1·5 cm above 97th centile<br>Head growth at least 1·5 cm per week for 2 weeks<br>Presence of any of the symptoms or signs of raised ICP	Vineland Social Maturity Scale	177
Libenson 1999	1986-1989	USA	Birth weight <1500g<br>Gestation <35 weeks<br>VI>97th centile+5mm<br>Raised ICP	Randomised mechanism not specified	Not specified	Serial Lumber Punctures (Daily or alternate days, until PHVD stabilized)	Acetazolamide (20mg/kg/d) and Frusemide (1mg/kg/d)	Rapidly progressive Hydrocephalus or severely increased ICP	None	16
Yapicioglu 2003	1999-2000	Turkey	Preterm infants with post-haemorrhagic hydrocephalus	Method of allocation unclear	Not specified	Daily lumbar Puncture (5–10 ml) +Streptokinase infusion at 0.5 ml/h for three days	Nil	No specified	None	12
Whitelaw 2007 and 2010	2003-2006	European	PHVD with VI >97th centile+4mm (or composite of other ventriculomegaly measures)	Computer generated random number; stratified by centre by variable blocks.<br>Opaque envelopes	Neuro-developmental measurement only	Fibrinolytics and irrigatration for 72 hours		VP shunt if, excessive head growth once weight was 2500g and CSF protein<1.5g/L	Bailey Scales of Infant Development II.	77
de Vries 2018	2006-2016	European	IVH grade 3 or 4<br>PHVD with VI between 97th and 97th+4mm<br>(or composite of other ventriculomegaly measures)	Computer generated random number; stratified by centre.	Nil	CSF tapping to maintain ventricular size	CSF tapping starting when/if VI expanded to 97th+4mm centile and AHW >10mm	Weight over 2000g<br>Protein <1.5g/L<br>RBC in CSF<100/mm3	None	126
